# Case Report: Presumed non-septic erosive sacroiliitis in a juvenile Bernese Mountain Dog: a 1.5-year follow-up

**DOI:** 10.3389/fvets.2025.1611436

**Published:** 2025-10-07

**Authors:** Johanna Mäkitaipale, Nele Eley

**Affiliations:** ^1^Department of Equine and Small Animal Medicine, Faculty of Veterinary Medicine, University of Helsinki, Helsinki, Finland; ^2^VetRad, Partnerschaftsgesellschaft, Gießen, Germany

**Keywords:** canine, sacroiliac joint, SI joint, sacroiliitis, erosive, pain

## Abstract

A four-month-old intact male Bernese Mountain Dog was presented for an orthopedic examination due to an abnormal hind limb gait and tarsal hyperextension for the past 2 weeks. Pain was observed upon palpation of the lumbosacral region. Moderate bilateral tarsal hyperextension and mild metatarsal outward rotation were observed. A wide, slightly abducted stance and a stiff gait were noted in the hind limbs. Radiographs revealed symmetric erosive lesions in the left sacroiliac joint (SIJ) and laxity of the hip joints. Computed tomography (CT) revealed multiple deep articular bone erosions with peripheral sclerosis of both SIJs, accentuating the iliac bones with consequential irregular widening of the sacroiliac joint spaces. The dog was treated conservatively with carprofen, physiotherapy, joint nutraceuticals, and exercise restriction. Complete resolution of clinical signs was observed on orthopedic examination within 3 weeks. The dog remained clinically normal, with partial resolution of the radiographic lesions, during a follow-up after 6 weeks. The dog remained in clinical remission, and radiographs and CT imaging revealed complete resolution of the erosive lesions at the 1.5-year follow-up. Sacroiliac joint pain is a possible rare cause of lower back pain, gait abnormality, and lameness in dogs. Case reports of both septic erosive and non-erosive, non-septic SIJ arthropathy have been described. However, a diagnosis of sterile erosive SIJ arthropathy is presumed in this case, since the clinical recovery was uneventful with carprofen and physiotherapy.

## Introduction

Sacroiliac joint (SIJ) pain is one of the most common causes of lower back pain in human beings, serving as the primary source of pain in approximately 25% of patients (1). Sacroiliitis, inflammation or infection of the sacroiliac joint, can be caused in humans by multiple diseases, including rheumatic inflammatory and non-inflammatory diseases, infectious diseases, and even malignancies (2). Case reports of septic sacroiliitis associated with bacterial infection have been reported in dogs, as well as a single case report of non-erosive, non-septic sacroiliitis in an adult dog (3–9). Degenerative changes, similar to those causing SIJ pain in humans, are commonly seen on computed tomography (CT) and magnetic resonance imaging (MRI) in dogs and are known to be inconsistent in their clinical significance (10–12). Clinical case reports of SIJ pain in dogs are rare. We report an unusual clinical case of presumed non-infectious, erosive sacroiliitis in a young puppy, which was managed with conservative treatment, resulting in full resolution of clinical signs.

## Case description

**Figure 1:**
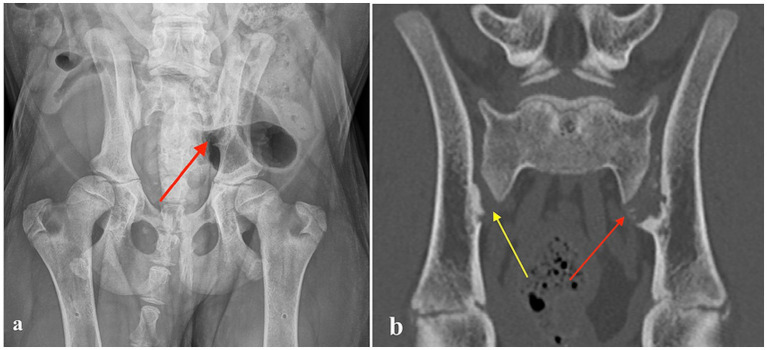
**(a)** Ventrodorsal radiograph of a four-month-old Bernese Mountain Dog with erosive lesions in the left sacroiliac joint (red arrow). **(b)** Computed tomography image (coronal plane) of the same dog, showing multiple small, randomly distributed cystoid and irregularly shaped concave defects within the left sacroiliac joint surface with deep peripheral sclerosis (left side red arrow). Similar but milder defects are visible on the right side (yellow arrow).

**Figure 2:**
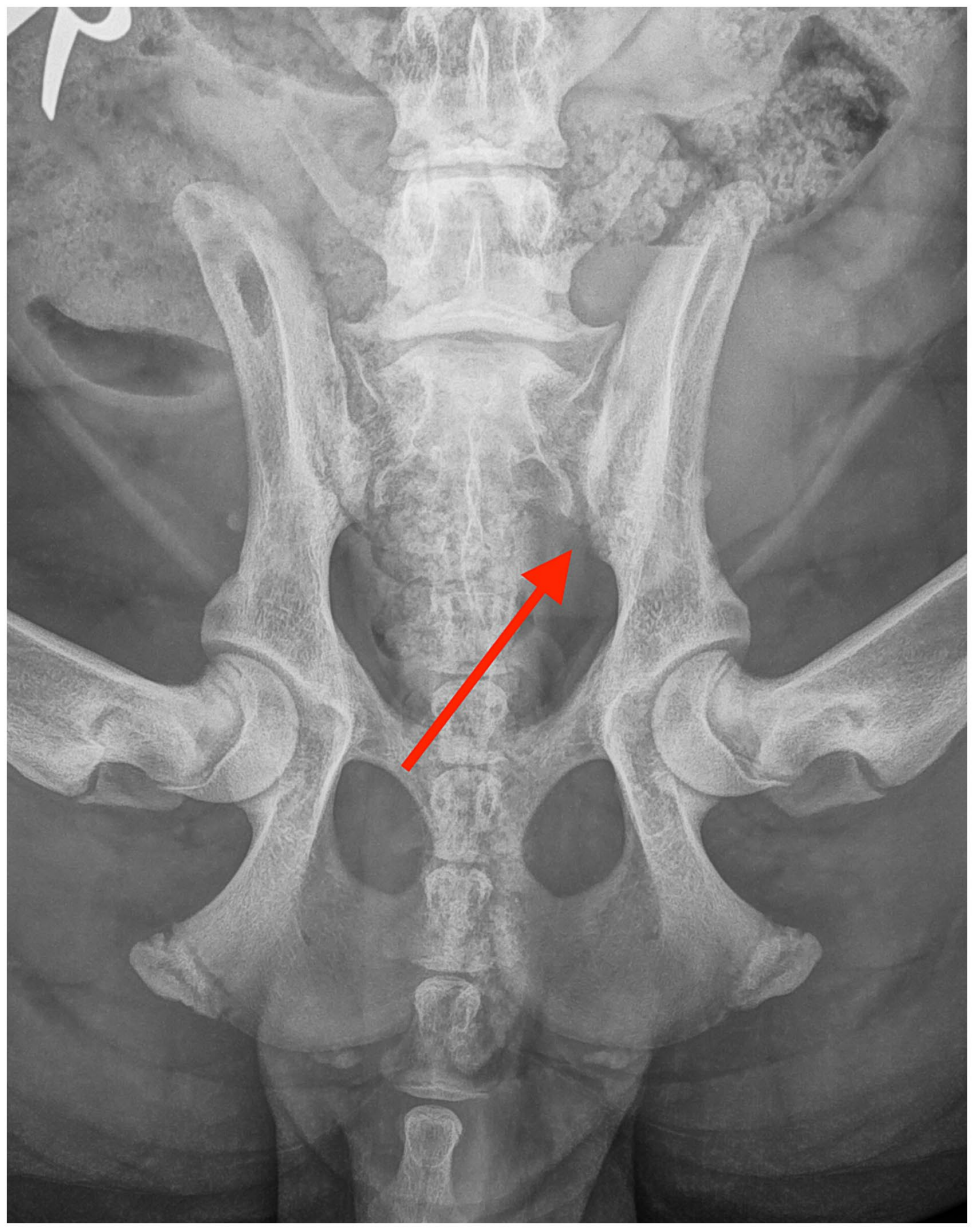
Ventrodorsal radiograph taken 6 weeks after the diagnosis, showing mild resolution of the erosive lesions in the sacroiliac joint (red arrow).

A four-month-old intact male Bernese Mountain Dog, weighing 20.8 kg, was presented for an orthopedic examination due to an abnormal hind limb gait and gradually worsening hyperextension of the right tarsus for the last 2 weeks. The dog had always been unwilling to jump onto the couch but otherwise moved normally. It was clinically healthy with no previous infections or predisposing illnesses. On orthopedic examination, palpation of the lumbosacral region elicited pain. The gait of the hind limbs was mildly stiff and a bit wide with some abduction. Moderate right tarsal hyperextension and mild left tarsal hyperextension, as well as mild metatarsal outward rotation, were also observed. The remainder of the orthopedic examination was unremarkable. The dog was sedated, and radiographs of the lumbar vertebrae and pelvis were obtained. The radiographs revealed erosive SIJ lesions on the left side and mild hip laxity (Figure 1). CT imaging was performed to obtain more precise information regarding the SIJ lesions. The CT study revealed multiple small, randomly distributed cystoid and irregularly shaped concave defects within the joint surfaces of both SIJs, with deep peripheral sclerosis (Figure 2b). Asymmetric widening of the SIJ spaces was also observed. The changes were severe on the left side and moderate on the right side. Both the radiographic and CT examinations also revealed changes in the coxofemoral joints consistent with mild canine hip dysplasia. The presumptive diagnosis for the clinical signs was inflammatory and infectious sacroiliitis. Degenerative and neoplastic diseases were considered less likely due to the patient’s age, clinical signs, and CT findings.

The dog received carprofen (4 mg/kg SID) for 3 weeks, physiotherapy, and joint nutraceuticals with glucosamine and chondroitin sulphate, and exercise was restricted to short leash walks. SIJ sampling to exclude bacterial growth was discussed but declined by the owner due to the rapid resolution of the clinical signs after the initiation of the treatment. In addition, juvenile pelvic symphysiolysis and double pelvic osteotomy (DPO) surgeries were discussed as possible treatments for hip laxity but were declined by the owner.

## Outcome

The patient was re-evaluated 3 weeks later. The dog was not lame and showed no pain on palpation of the lumbosacral or SIJ areas. Extension of the left hip was mildly restricted compared to the right hip. Metatarsal outward rotation was mild. Otherwise, the orthopedic examination was unremarkable. Physiotherapy was continued, and carprofen was administered at 2 mg/kg once daily for one more week. After a month, the dog was clinically normal. Metatarsal outward rotation was the same as before. The recheck radiographs after 6 weeks revealed a significant reduction in the size of the erosive changes, with an overall increase in bone opacity and reduced width of the SIJ spaces (Figure 2). The resolution of the radiographic changes was incomplete at this point.

**Figure 3:**
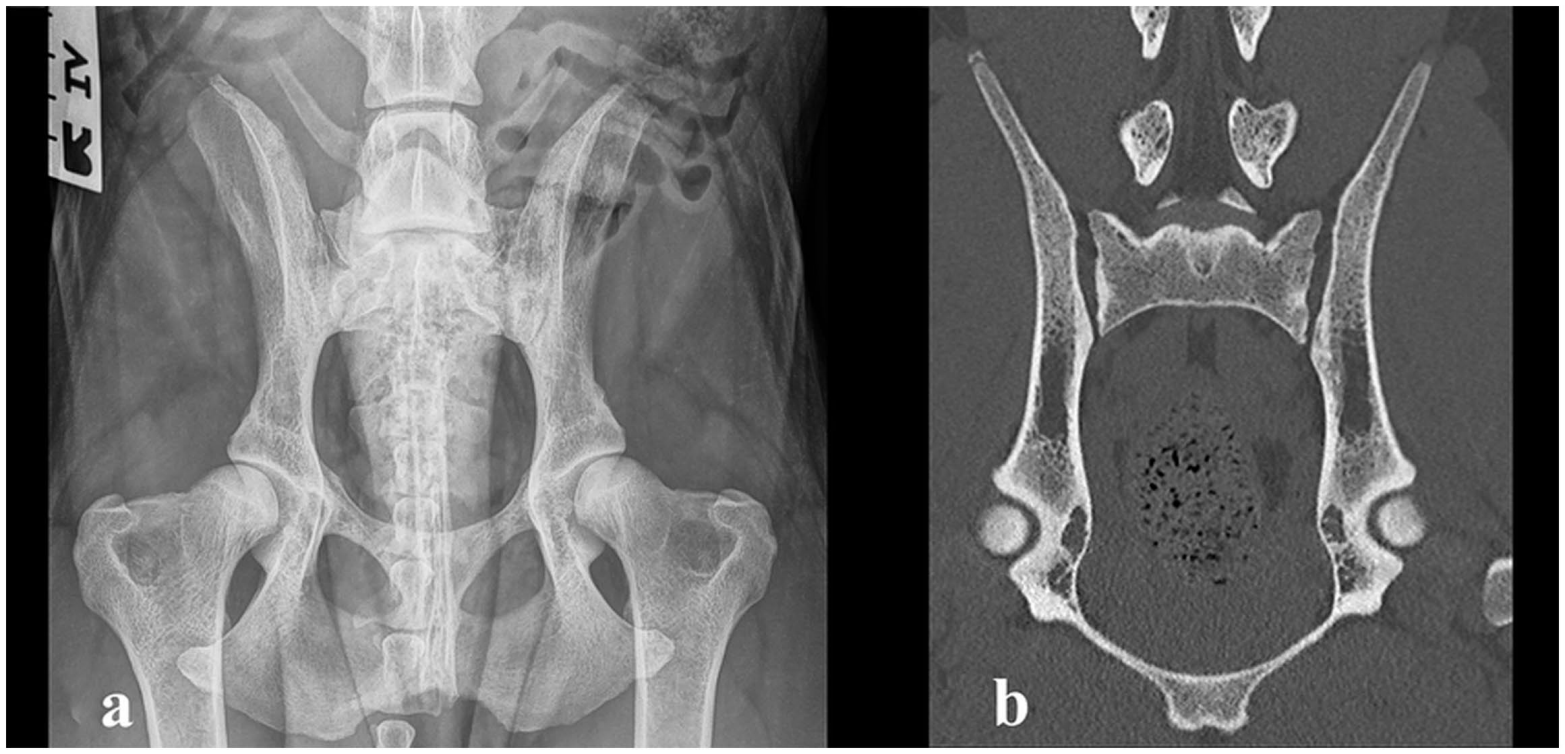
**(a)** Ventrodorsal radiograph taken 1.5 years after the diagnosis, showing complete resolution of the erosive changes in the sacroiliac joint and moderate hip dysplasia. **(b)** Computed tomography image (coronal plane) of the sacroiliac joint 1.5 years after the diagnosis, showing complete resolution of the changes.

Physiotherapy was continued, and the dog was allowed to return to normal activity. After 1.5 years, the owner reported that the dog maintained normal activity without clinical complaints. Recheck radiography and CT were performed during the official hip and elbow dysplasia screening. Palpation of the SIJs and lumbosacral region was non-painful. The range of motion of the hips was normal and non-painful. Mild tarsal outward rotation was observed clinically, but the orthopedic examination was otherwise unremarkable. The radiographic and CT images revealed no evidence of erosive or sclerotic lesions, and the appearance of the SIJs was considered within normal limits (Figure 3). In the official Fédération Cynologique Internationale hip dysplasia screening, the hips were classified as D/D (moderate hip dysplasia).

The clinical and radiographic progression in our patient strongly supported a diagnosis of non-septic erosive sacroiliitis. However, definitive exclusion of infection was not possible due to the absence of joint aspiration and culture.

## Discussion

Spontaneous non-septic erosive sacroiliitis was diagnosed as the presumed cause of the clinical signs and imaging changes in a juvenile Bernese Mountain Dog. The sacroiliac joint has a complex synovial and fibrocartilaginous anatomy with limited motion. It plays a major role in weight-bearing and the transmission of propulsion from the pelvic limbs to the spine. The SI joint is thoroughly innervated, and pain and dysfunction are common in humans (1). Degenerative SI joint disease, known as ankylosis capsularis ossea, has been frequently reported in diagnostic imaging, especially in large- and giant-breed dogs (10, 12–14). An imbalance between increasing body weight and the size of the SI joint contact area is a main reason for this. Force exerted on the SI joint in large-breed dogs is twice as high as that in toy breeds (15). Chronic overuse, trauma, and microtrauma from daily repetitive activities are suspected to cause stress to the supporting ligaments, leading to laxity SIJ (15).

Individual case reports have reported septic and non-septic non-erosive sacroiliitis in dogs (3–9). However, we are not aware of previous reports of clinically significant non-septic erosive SIJ arthropathy in dogs. In humans, many diseases, including rheumatic inflammatory and non-inflammatory diseases, infectious diseases, and even malignancies, can cause sacroiliitis (2). Erosive sacroiliitis is common in children with the enthesitis-related spondyloarthritis subtype of rheumatoid juvenile idiopathic arthritis (16, 17), but rheumatoid diseases in juvenile dogs are uncommon. In a case report of non-erosive, non-septic sacroiliitis in an adult German Shepherd, trauma or a paraneoplastic syndrome were suspected as possible causes of the disease (9). The etiology of non-septic sacroiliitis is so far unknown in dogs. An inflammatory or self-limiting autoimmune cause might provide an explanation for the disease in our patient.

We were unable to perform sampling of the SIJ for a final diagnosis and to rule out septic sacroiliitis due to a lack of owner consent. This is one limitation of our case report. However, the dog responded quickly and well to conservative management with non-steroidal anti-inflammatory medication within a few days, making bacterial infection unlikely. The imaging findings resolved at a slower pace but consistently, and no clinical relapse occurred during the 1.5-year follow-up. Therefore, rheumatoid or other underlying chronic predisposing diseases were considered unlikely.

The dog had mild bilateral hip laxity, which is a differential diagnosis for an abnormal gait during growth and pain, but it was not considered the primary cause of the clinical signs in this case. The clinical signs resolved with conservative treatment, and the dog was nearly normal at the 3-week recheck and fully normal at 6 weeks, despite the mild concurrent hip dysplasia. Juvenile pelvic symphysiolysis and DPO surgeries were discussed to reduce the risk of hip dysplasia, but the owner opted for total hip replacement if needed in the future. At the 1.5-year follow-up visit, moderate hip dysplasia was diagnosed; however, the dog was clinically normal, so the owner elected conservative treatment with physiotherapy instead of surgical treatment at that time. Mild metatarsal outward rotation was observed on orthopedic examination, and breed-specific tarsal malformation was suspected to be the cause (18). As the rotation was mild, further diagnostics were not performed to confirm the diagnosis.

Sacroiliac joint (SIJ) pain can mimic pain originating from the hip joints and lumbar spine, causing challenges in diagnosis (1). Several diagnostic provocation maneuvers exist for humans, but intra-articular local anesthetic blocks are often needed for precise diagnosis (1). In dogs, SIJ pain is probably highly underdiagnosed due to being overlooked and the challenges associated with diagnostics. A list of clinical diagnostic tests for the recognition of SI pain and dysfunction in dogs has been described (19). As the accuracy of these provocative tests may be only fair, similar to humans, further studies are needed to evaluate the use of intra-articular local anesthetic blocks for the diagnosis and treatment of SI pain in dogs. In this case, the exact localization of the pain at the first visit was difficult as the dog was sensitive throughout the whole lumbosacral area. Hip dysplasia and osteochondrosis of the sacrum or L7 vertebrae were considered possible primary differential diagnoses before diagnostic imaging revealed SIJ lesions.

Due to the complex synovial and fibrocartilaginous anatomy of the SIJ, CT imaging, which was available at the hospital, was performed to get better visualization of the SIJs. Contrast media was not used, which is another limitation of our case report. Imaging changes can lag behind clinical signs in X-ray-based modalities such as standard radiography and CT, since it takes time for osseous turnover in mineral bone density to become visually apparent. Cross-sectional imaging is the preferred method for advanced SIJ diagnostics and has been validated for recognizing degenerative SIJ lesions in dogs (9). CT and MRI have been used to diagnose canine SIJ lesions and infectious sacroiliitis (4, 7, 12). In humans, both modalities are used. Especially, in human patients with spondyloarthropathy, MRI is preferred due to its better visualization of bone marrow edema, which is an early sign of inflammation (2). While CT is highly sensitive for detecting cortical bone changes and subtle erosions, certain limitations compared to MRI should be acknowledged. MRI offers superior contrast resolution for assessing early inflammatory changes, including bone marrow edema, synovitis, and periarticular soft tissue involvement, which may precede overt osseous erosion visible on CT. Consequently, MRI can detect inflammatory arthropathy at an earlier stage. However, in cases where the disease has progressed to erosive changes, the diagnostic performance of CT is comparable, particularly for evaluating the extent and morphology of cortical erosions. Moreover, CT provides excellent spatial resolution, is less susceptible to motion artefacts, and allows for rapid acquisition. Therefore, while MRI may offer incremental information regarding early or predominantly soft tissue disease, CT remains a robust and reliable tool for the assessment of erosive arthropathy, especially when MRI is unavailable or contraindicated.

The pathomechanism of non-septic SIJ disease in dogs is poorly understood at this point. Nevertheless, sacroiliitis and other SIJ pathologies in dogs seem to have gained increasing recognition and significance in clinical veterinary medicine and scientific literature in recent years. Erosive arthritis can usually be divided into septic and non-septic arthritis. The observed clinical evolution of our patient, in conjunction with the characteristic radiographic progression, was highly consistent with non-septic erosive sacroiliitis and provided strong supportive evidence for this diagnosis. Nevertheless, the possibility of an infectious cause was not conclusively ruled out, given that joint aspiration and microbiological culture—the gold-standard methods for confirming or excluding septic involvement—were not performed. Erosive non-septic sacroiliitis should be considered in diagnosing and treating dogs with lower back pain.

## Data Availability

The original contributions presented in the study are included in the article/supplementary material, further inquiries can be directed to the corresponding author.
